# Automated Classification and Segmentation in Colorectal Images Based on Self-Paced Transfer Network

**DOI:** 10.1155/2021/6683931

**Published:** 2021-01-20

**Authors:** Yao Yao, Shuiping Gou, Ru Tian, Xiangrong Zhang, Shuixiang He

**Affiliations:** ^1^School of Artificial Intelligence, Xidian University, Xi'an, Shanxi 710071, China; ^2^School of Information Engineering, Hangzhou Vocational and Technical College, Hangzhou, Zhejiang 310018, China; ^3^Department of Gastroenterology, First Affiliated Hospital of Xi'an Jiaotong University, Xi'an, Shanxi 710071, China

## Abstract

Colorectal imaging improves on diagnosis of colorectal diseases by providing colorectal images. Manual diagnosis of colorectal disease is labor-intensive and time-consuming. In this paper, we present a method for automatic colorectal disease classification and segmentation. Because of label unbalanced and difficult colorectal data, the classification based on self-paced transfer VGG network (STVGG) is proposed. ImageNet pretraining network parameters are transferred to VGG network with training colorectal data to acquire good initial network performance. And self-paced learning is used to optimize the network so that the classification performance of label unbalanced and difficult samples is improved. In order to assist the colonoscopist to accurately determine whether the polyp needs surgical resection, feature of trained STVGG model is shared to Unet segmentation network as the encoder part and to avoid repeat learning of polyp segmentation model. The experimental results on 3061 colorectal images illustrated that the proposed method obtained higher classification accuracy (96%) and segmentation performance compared with a few other methods. The polyp can be segmented accurately from around tissues by the proposed method. The segmentation results underpin the potential of deep learning methods for assisting colonoscopist in identifying polyps and enabling timely resection of these polyps at an early stage.

## 1. Introduction

The International Agency for Research on Cancer released research data on global cancer status in 2018. The report reported the incidence and mortality of 36 types of tumors in 185 countries around the world, comprehensively. The data showed that the incidence of colorectal cancer ranked third (10.2%), and the mortality rate ranked second (9.2%) [1]. As we known, the mortality rate of colorectal cancer can be reduced significantly by early removal of polyps [2] which can be found according to the early screening. Colorectal polyp, a benign disease, has specific imaging characteristics such as shape or surface structure and color [3]. Colorectal colonoscopy is the main method of diagnosing intestinal diseases. With a great number of colorectal images, the microscopic examination presents labor-intensive and time-consuming problems [4]. In addition, the pathological diagnosis of colonoscopy biopsy samples is prone to deviations due to individual pathologists' experience and knowledge [5]. The accuracy of diagnosis depends on the experience of the microscopy doctor, and the difference in diagnosis accuracy between experienced doctors and less experienced doctors is greater than 10%. Therefore, it is necessary to distinguish polyps from normal tissue and tumor using colorectal optical images.

However, it is difficult for the diagnosis of colorectal optical images. Firstly, the low light and interference of liquid often result in poor imaging quality of colorectal images. Secondly, the edges of normal tissue and polyp types are blurred. It causes the classification accuracy of normal tissue, polyp, and tumor to be low. Thirdly, the individual differences of polyps are mainly manifested in shape, color, and surface contour for polyps. And colorectal polyps are heterogeneous resulted that the segmentation of polyp becomes challenging.

Previous research showed that deep learning has given good results in medical images processing, such as tumor detection, classification, segmentation, retrieval, and prediction, especially for diagnosis and treatment of the brain [6, 7], breast [8], lung [9, 10], gastric [11], prostate cancers [12, 13], and histopathology [14]. Meanwhile, endoscopy-assisted diagnosis has also made some progress using deep learning, especially in colorectal endoscopy. There are two types for colorectal image detection: pathology and optical colonoscopy images. Here is the introduction to the image diagnosis progress.

*For the pathology colorectal images*, the recent advancement of deep learning is adapted. Thakur et al. [15] reviewed the development of an AI system in CRC pathology image analysis using deep learning. Korbar et al. [16] proposed an automatic image-understanding method to help pathologists with histopathological characterization and diagnosis of colorectal polyps. Sena et al. [17] propose a deep learning approach to recognize four different stages of cancerous tissue development. Lizuka et al. [18] trained convolutional neural networks (CNNs) and recurrent neural networks (RNNs) on biopsy histopathology whole-slide images (WSIs) of stomach and colon*. For the optical colorectal images*, *there are lots of researches on* detection and segmentation of colorectal polyps. Some methods take into account time series: Urban et al. [19] used deep learning to localize and identify polyps in real time with 96% accuracy in screening colonoscopy. Klare et al. [20] proposed the APDS with which the colonoscopy system of the video stream is captured by a frame-grabber device in HD. Wang et al. [21] used real-time automatic detection system to increase colonoscopic polyp and adenoma detection rates; some methods take into account spatial information: Li et al. [22] used a fully convolutional neural network structure for segmenting colorectal polyps. Yang et al. [23] developed convolutional neural network (CNN) models which automatically categorized colorectal lesions into several stages ranging from nonneoplastic lesions to advanced CRC with conventional white-light colonoscopy images. Zhang et al. [24] developed a fully automatic algorithm to detect and classify hyperplastic and adenomatous colorectal polyps. Others are from the semantic information: Wickstrom and Kampffmeyer [25] proposed a novel method for estimating the uncertainty associated with important features in the input and demonstrated how interpretability and uncertainty can be modeled for semantic segmentation of colorectal polyps. The above colorectal image processing methods using deep learning have achieved good performance.

Based on the above analysis of colonic *pathology and optical* colorectal image literature, deep learning methods are proposed on detection or segmentation of colorectal polyps. However, unlike the recent research based on single task, our method takes into account multitask: colorectal image classification and polyp image segmentation. In the proposed STVGG, transfer learning and self-paced learning are used to solve the unbalance and the difficult sample learning. STVGG transfers ImageNet network parameters to VGG network and calculates the loss value of each image in the forward propagation with the age parameter. In addition, the trained STVGG model of colorectal classification is shared to Unet segmentation model to deal with distinguishing polyp and normal tissues.

## 2. Materials and Methods

### 2.1. Data Acquisition and Preprocessing

A total of 50 patients were examined under colonoscopies, and images were collected from the anorectal department of a hospital in Shaanxi Province, China, under ethical approval. Three experienced endoscopists were invited to classify the normal tissue, polyp, and tumor, and the ground truth was acquired. The data preprocessing was as follows:

*Firstly*, *data filtering*: uncleaned or unclear colorectal images were removed. After image filtering, the set of endoscopic images consisted of 487, 1374, and 1200 images with normal tissue, polyp, and tumor, respectively, taken under either white light (WL) or narrow band imaging (NBI) endoscopy.

*Secondly*, *dataset split*: the data were divided into training set, validation set, and test set according to the ratio of 2 : 1 : 2.

*Thirdly*, *data argumentation*: the argumentation methods were rotation, flip, translation, and cropping. The training set and validation set were argumentized by four times

*Finally*, *data resizing*: the data was resized to 440 × 440 × 3 to maintain the integrity of the intestinal wall.

### 2.2. Automatic Classification in Colorectal Endoscopy Based on STVGG

Because the performance of training network is poor by using colorectal images directly, Network pretrained on ImageNet is introduced to obtain a good classification result. Meanwhile, polyp areas are more difficult to be classified than normal tissue and tumor. This paper introduces self-paced regularization items to assign different sample weights for training samples. Self-paced learning injects the difficulty metric into the optimization model and updates the model parameters based on the current sample ordering and the metric based on the learning effect. It obtains a new round of difficulty ordering of samples and finally achieves the purpose of adaptive sample ordering.

**Figure 1:**
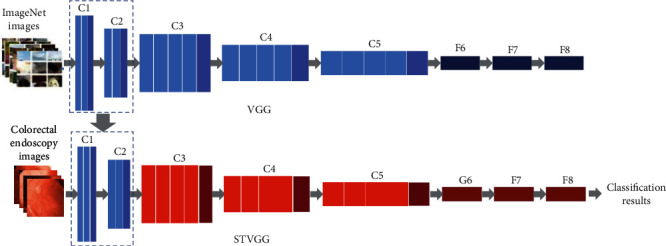
The overview of STVGG classification method.

In our method, in order to fully use data of ImageNet, the parameters of *C*_1_ and *C*_2_ from pretrained VGG19 model on ImageNet are transferred to STVGG. And the practical colorectal images are used as training data to update other layer parameters of the STVGG model. The self-paced learning algorithm is introduced to STVGG for dealing with those difficult and unbalance samples to improve classification performance. The overview of STVGG classification method is shown in Figure 1, where *C*_*i*_ represents the *i*^th^ convolutional patch, *F* represents the fully connected layer, and *G* represents the global average pooling layer. In this study, the first fully connected layer *F*_6_ of VGG19 is replaced with the global average pooling layer *G*_6_ to reduce the amount of model parameters and prevent overfitting and to get the pretrained model with the parameters of *C*_1_ and *C*_2_ layers.

The cross-entropy is selected as loss function *l*(*y*_*i*_, *g*(**X**_*i*_)). Forward propagation is used to calculate the loss *l* in STVGG network:

(1) $$\begin{array}{c} l\left(y_{i},g\left(X_{i}\right)\right)=-\overset{c}{\underset{j=1}{\sum }}I\left\{y_{i}=j\right\}\log\left(\frac{e^{\omega_{j}^{T}z_{i}^{l}}}{\sum_{k=1}^{c}e^{\omega_{k}^{T}z_{i}^{l}}}\right). \end{array}$$

In the Eq. (1), *c* is the number of disease categories in the colorectal endoscopy dataset, and **ω**_*j*_^T^ is the weight parameter of the *j*^th^ output node. **z**_*i*_^*l*^ represents the output vector in the last fully connected layer of the network. *I*{*y*_*i*_ = *j*}∈{0, 1}, when the predicted result of the sample is consistent with the label *I* = 1, otherwise *I* = 0.

Furthermore, self-paced learning is used to modify the STVGG network. The network objective function is rewritten as follows:

(2) $$\begin{array}{c} \underset{\omega ,b}{\min}\;E_{\text{spl}}\left(w,b\right)=\underset{\omega ,b}{\min}\left[\frac{1}{n}\overset{n}{\underset{i=1}{\sum }}v_{i}\cdot l\left(y_{i},g\left(X_{i}\right)\right)+f\left(v_{i},\lambda \right)\right] \end{array}$$

Parameters *w* and *b* are the weight and bias of the STVGG network, respectively, **v** = [*v*_1_ ⋯ *v*_*i*_ ⋯ *v*_*n*_] represents the weight of *n* samples, and *f*(*v*_*i*_, *λ*) is the binary self-paced regular term defined in Eq. (3).

(3) $$\begin{array}{c} f^{H}\left(v_{\text{i}},\lambda \right)=-\lambda v_{i};v_{i}*\left(l,\lambda \right)=\left\{\begin{array}{c} 1\text{ if }l<\lambda ,\\ 0\text{ if }l\geq \lambda . \end{array}\right. \end{array}$$

*w*, *b*, and **v** of the STVGG network are optimized by iteration until the model converges to get a good classification network. Flowchart of STVGG algorithm is shown in Algorithm 1.

### 2.3. Automatic Segmentation in Polyp Image

**Figure 2:**
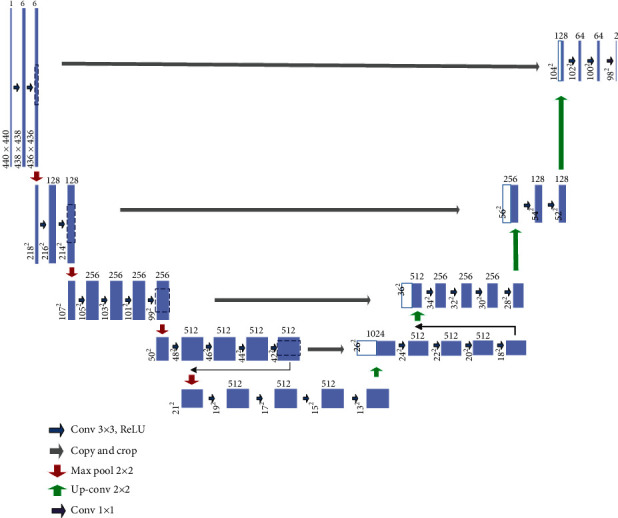
The framework of our segmentation model.

After classification task is completed, the parameters *C*_1_ − *C*_5_ of the trained STVGG in the colorectal endoscopy classification task is shared to the segmentation task as the code part, while the Unet network framework is used in colorectal endoscopy segmentation task. And the decoding part of the original Unet is also adjusted with the corresponding encode part. Compared with the original Unet, the channel number of downsampling in the last layer is not increased for the proposed model. The framework of our segmentation model is shown in Figure 2.

Each rectangular box corresponds to a multichannel feature map. The number on the left side of the rectangular box indicates the size of each channel of the feature map. The number at the top of the rectangle indicates the channel number in the feature map. The blue, red, green, and purple arrows indicate the convolution operation with a convolution kernel size of 3 × 3, the max pooling with stride of 2 × 2, and the upsampling and the convolution with a convolution kernel size of 1 × 1, respectively. The gray arrow indicates that the feature map of the encoding part is cropped and copied with the feature map of the decoding part.

### 2.4. Comparison with Other Methods

The selection of comparison methods is based on the baseline VGG model adding some training strategies, and the specific strategies are as follows:

(1) VGG19 with transfer learning strategies (VGG+TL)

The parameters *C*_1_ − *C*_2_ in VGG19 are transferred from ImageNet network to extract low-level features well shared with natural images in colorectal endoscopy images.

(2) VGG19 with the strategy of structure retention color normalization (VGG+SRCN)

The data are collected from different periods, different patients, and equipment in different periods. Therefore, SRCN strategy is used so that color features of processed image tend to be consistent and reduce intraclass differences.

(3) VGG19 with strategy of spatial pyramid pooling (VGG+SPP)

SPP [26] layer on the VGG19 network is adopted, and it obtains a fixed length feature vector to aggregate the features and avoid geometric distortion in feature maps.

### 2.5. Evaluation of the Classification and Segmentation

The segmentation results are evaluated both visually and quantitatively, given the ground truth, our classification and segmentation results. The segmentation performance is evaluated by these evaluation metrics: accuracy, sensitivity, specificity, and dice similarity coefficient (DSC). We use TP, FP, TN, and FN to represent true positive, false positive, true negative, and false negative. And *L*_1_ and *L*_2_ represent the manual annotation and our method segmentation results, respectively, and these indexes are defined as follows:

(4) $$\begin{array}{c} \text{Accuracy}=\frac{\text{TP}+\text{TN}}{\text{TP}+\text{TN}+\text{FP}+\text{FN}}, \end{array}$$

(5) $$\begin{array}{c} \text{Sensitivity}=\frac{\;|\;L_{1}\cap L_{2}\;|\;}{\;|\;L_{1}\;|\;}, \end{array}$$

(6) $$\begin{array}{c} \text{Specificity}=\frac{\;|\;\sim \left(L_{1}\;|\;L_{2}\right)\;|\;}{\;|\;\sim L_{1}\;|\;}, \end{array}$$

(7) $$\begin{array}{c} \text{DSC}=\frac{2\;|\;L_{1}\cap L_{2}\;|\;}{\left(\;|\;L_{1}\;|\;+\;|\;L_{2}\;|\;\right)}. \end{array}$$

## 3. Experimental Results and Discussion

In this paper, the experimental environment was set in Python3.6.0, Tensorflow-GPU 1.7.0, Keras 2.1.3, SimpleITK 0.8.1, Nvidia Titan Pascal GPU (1080 Titan), and Cudnn V9.0. Verified by experiment, the colorectal image size of 440 was superior to 330 and 540. The average accuracy of the final test set was 0.03 and 0.01 higher than the latter two, respectively. The colorectal data with original image size range of 228 to 586 was resized to 440 × 440.

*Choosing optimization functions*. Experiments showed that the average classification accuracy of SGD is 0.02 and 0.05 higher than RmSprop and Adam, respectively. Therefore, SGD was finally selected as our optimization function.

*Selecting transfer layers*. Experiments froze the parameters of the 4, 8, 12, and 16 layers, respectively. It showed that when the parameters of the first 4 layers were frozen, the best average classification accuracy was achieved.

*Cross-entropy are chosen as loss function*. The relevant parameters were as follows: learning rate is 0.0001, decay = 1*e* − 6, and the parameter of Nesterov Momentum was set to 0.9. The batch size was 8. *λ* was initialized to 1.1, and the updating step was 0.05. As training began, *λ* became larger and the tolerance of difficult samples was greater.

### 3.1. Colorectal Endoscopy Image Classification

The colorectal endoscopy image classification accuracy is shown in Table 1. It can be seen from Table 1 that the polyp accuracy of VGG network was the lowest. The classification accuracies of VGG+SRCN on polyps and normal tissue are improved as it could decrease the intraclass differences. VGG+SPP also improved the classification accuracies of normal tissue and colorectal polyp but polyp accuracy was relatively low because the edges of normal tissue and polyp types are blurred. As an improvement strategy, the accuracy of VGG+TL was also improved. But compared with strategies of SRVN and SPP, the effect is not significant. In this study, STVGG was proposed and the experimental results showed that the overall accuracies were greatly improved.

The main reason is that STVGG can classify difficult-to-classify samples, for example, the small inflammatory or hyperplastic polyps which are very similar to normal colonoscopy images, and the polyps with ulcers, large areas of bleeding, and reticulated polyps, which are closer to the characteristics of tumor. The STVGG method can significantly improve the accuracy of polyp under the condition of ensuring the classification accuracy of tumor and normal ones, and the method converges in about 10 generations of training.

### 3.2. Polyp Image Segmentation

**Figure 3:**
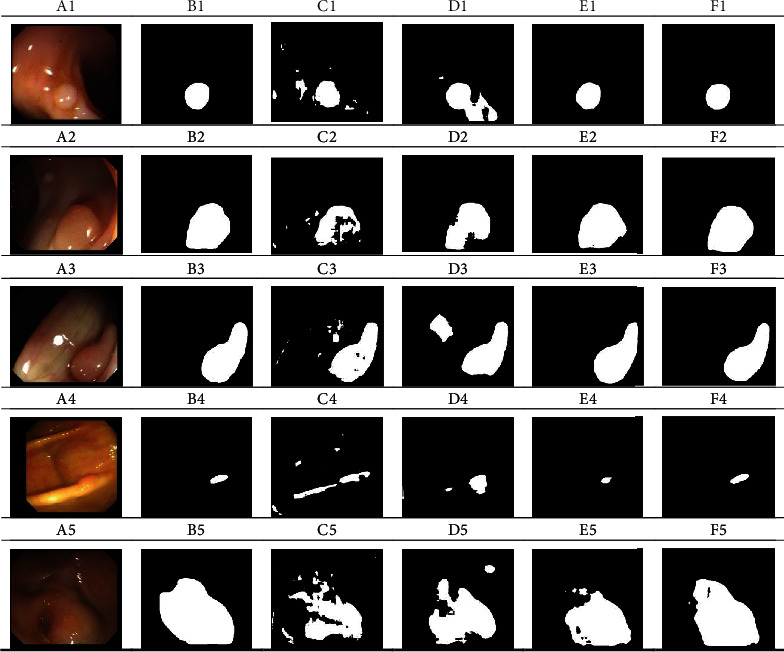
Segmentation in colorectal endoscopy images. (Ai) Original images, (Bi) ground truth, (Ci) Segnet, (Di) Unet, (Ei) TLVGG, and (Fi) ours.

In the above colorectal endoscopy image classification task, a relatively good classification result was obtained by STVGG model. Therefore, polyp segmentation was designed based on classification task. Doctors usually used polyp's images to make a decision whether surgical resection is required based on pathological diagnosis.  Figure 3 shows five sequences of polyp images. (Ai) is the original image, (Bi) is ground truth, (Ci) is the segmentation result of Segnet network, (Di) is the segmentation result of Unet network, (Ei) is the segmentation result of TLVGG network, and (Fi) is the segmentation result of STVGG network.

Figure 3 indicates that the results of Segnet method are greatly affected by the surrounding environment, and the segmentation result is not good. The results of Unet method are more superior than those of Segnet in big target but the effect is not obvious. Compared to those methods, the results of TLVGG method made great progress especially in surrounding and small target, but the segmentation results of large targets are not ideal. Our method shows the best results, no matter it is segmentation of large and small objects or environmental interference.

Table 2 shows that the Segnet segmentation method does not segment the polyp in its complete shape. Using Unet for segmentation of polyps is accurate, but oversegmentation is also obvious. The Unet is more sensitive to light spots in the imaging process, and it is easy to treat the reflective part as a polyp.

The segmentation performances of the TLVGG network were obviously better than Segnet and Unet. The segmentation target contour was close to the real target, but there were still missed detections. The STVGG model worked best because ImageNet network parameters were transferred to VGG network to acquire good initial network. And self-paced learning was used to optimize the network so that the classification performance of label unbalanced samples was improved.

## 4. Conclusions

To address this issue of label unbalanced and difficult colorectal data, we presented an automatic processing pipeline for classification and segmentation based on colorectal images. STVGG network used transfer learning and self-paced learning in order to acquire good initial network and solve the problem of label unbalanced and difficult sample classification. And then STVGG network was shared as the encoding part of Unet as encoder of the segmentation task, and image segmentation task was achieved. The experimental illustrated that the proposed method obtained higher classification accuracy (96%) and segmentation performance compared with other a few methods. This proposed method may be applied to other image researches, such as stomach, ear, nose, and throat. Possible future improvements can be made in parameter adaptation.

## Figures and Tables

**Algorithm 1:**
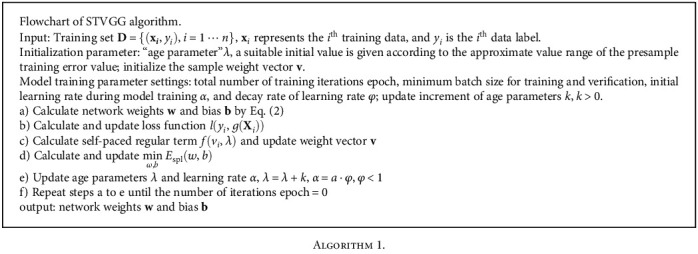
Algorithm 1.

**Table 1:** Classification accuracy obtained for different methods.

Category	VGG	VGG+SRCN	VGG+SPP	VGG+TL	STVGG
Tumor	0.98	0.98	0.94	0.96	0.98
Normal tissue	0.90	0.94	0.99	0.95	0.99
Polyp	0.7	0.84	0.91	0.89	0.95
Average accuracy	0.76	0.87	0.93	0.91	0.96

**Table 2:** Segmentation indexes obtained from different methods.

	Segnet	Unet	TLVGG	STVGG
DSC	0.6210 ± 0.2370	0.6980 ± 0.3005	0.8267 ± 0.2066	0.8455 ± 0.2030
Sen	0.6916 ± 0.2677	0.7591 ± 0.3317	0.8222 ± 0.2462	0.8323 ± 0.2201
Spe	0.9766 ± 0.0180	0.9834 ± 0.0235	0.9933 ± 0.0095	0.9949 ± 0.0067

## Data Availability

The data used in the article are from the anorectal department of a hospital in Shaanxi Province, China, under ethical approval.
